# Cohort Profile: The United Kingdom Research study into Ethnicity and COVID-19 outcomes in Healthcare workers (UK-REACH)

**DOI:** 10.1093/ije/dyac171

**Published:** 2022-08-27

**Authors:** Luke Bryant, Robert C Free, Katherine Woolf, Carl Melbourne, Anna L Guyatt, Catherine John, Amit Gupta, Laura J Gray, Laura Nellums, Christopher A Martin, I Chris McManus, Claire Garwood, Vishant Modhawdia, Sue Carr, Louise V Wain, Martin D Tobin, Kamlesh Khunti, Ibrahim Akubakar, Manish Pareek, UK-REACH Collaborative Group+, Manish Pareek, Laura Gray, Laura Nellums, Anna L Guyatt, Catherine John, I Chris McManus, Katherine Woolf, Ibrahim Akubakar, Amit Gupta, Keith R Abrams, Martin D Tobin, Louise Wain, Sue Carr, Edward Dove, Kamlesh Khunti, David Ford, Robert Free

**Affiliations:** Department of Respiratory Sciences, University of Leicester, Leicester, UK; Department of Respiratory Sciences, University of Leicester, Leicester, UK; NIHR Leicester Biomedical Research Centre, University of Leicester, Leicester, UK; University College London Medical School, London, UK; Genetic Epidemiology Research Group, Department of Health Sciences, University of Leicester, Leicester, UK; Genetic Epidemiology Research Group, Department of Health Sciences, University of Leicester, Leicester, UK; Genetic Epidemiology Research Group, Department of Health Sciences, University of Leicester, Leicester, UK; Oxford Teaching Hospitals NHS Foundation Trust, Oxford, UK; Biostatistics research group, Department of Health Sciences, University of Leicester, Leicester, UK; Population and Lifespan Sciences, School of Medicine, University of Nottingham, Nottingham, UK; Department of Respiratory Sciences, University of Leicester, Leicester, UK; Department of Infection and HIV Medicine, University Hospitals of Leicester NHS Trust, Leicester, UK; University College London Medical School, London, UK; Department of Respiratory Sciences, University of Leicester, Leicester, UK; Department of Respiratory Sciences, University of Leicester, Leicester, UK; General Medical Council, London, UK; Department of Nephrology, University Hospitals of Leicester NHS Trust, Leicester, UK; Genetic Epidemiology Research Group, Department of Health Sciences, University of Leicester, Leicester, UK; Genetic Epidemiology Research Group, Department of Health Sciences, University of Leicester, Leicester, UK; Department of Cardiovascular Sciences, University of Leicester, Leicester, UK; Faculty of Pop Health Sciences, School of Life & Medical Sciences, University College London, London, UK; Department of Respiratory Sciences, University of Leicester, Leicester, UK; Department of Infection and HIV Medicine, University Hospitals of Leicester NHS Trust, Leicester, UK

Key Features

- The UK-REACH cohort was established to understand why ethnic minority healthcare workers (HCWs) are at risk of poorer outcomes from COVID-19 when compared with their White ethnic counterparts in the UK. Through study design, it contains a uniquely high percentage of participants from ethnic minority backgrounds about whom a wide range of qualitative and quantitative data have been collected.
- A total of 17 891 HCWs aged 16–89 years (mean age: 44) have been recruited from across the UK via all major healthcare regulators, individual National Health Service hospital trusts and UK HCW membership bodies who advertised the study to their registrants/staff to encourage participation in the study.
- Data available include linked healthcare records for 25 years from the date of consent and consent to obtain genomic sequencing data collected via saliva. Online questionnaires include information on demographics, COVID-19 exposures at work and home, redeployment in the workforce due to COVID-19, mental health measures, workforce attrition and opinions on COVID-19 vaccines, with baseline (*n* = 15 119), 6 (*n* = 5632) and 12-month follow-up (*n* = 6535) data captured.
- Request data access and collaborations by following documentation found at https://www.uk-reach.org/main/data_sharing.

## Why was the cohort set up?

UK-REACH is a UK-wide prospective cohort established in November 2020 in response to the COVID-19 pandemic.^1^ COVID-19 has spread rapidly across the world, causing significant levels of morbidity and mortality, and devastating health economies in many countries. Healthcare workers (HCWs) have been at the forefront of the response to the pandemic and thus have been identified as being at increased risk of infection by SARS-CoV-2 and associated adverse outcomes.^2–4^ Furthermore, a number of studies have indicated that this risk of infection and adverse outcomes is greater for individuals from ethnic minority groups, particularly when compared with White HCWs.^3^ Emerging evidence also suggests that ethnic minority groups may be at an increased risk of long-term COVID-19 sequelae and poor mental health outcomes such as anxiety, depression and post-traumatic stress.^5^^,^^6^

The quality of data related to COVID-19 risk and outcomes in HCWs is relatively poor, with very few large-scale representative studies in clinical or ancillary HCWs in healthcare settings stratified by ethnicity or occupation type, once potential confounders have been controlled for. The UK-REACH longitudinal cohort aims to address this disparity by examining differences in COVID-19 clinical outcomes [diagnosis, hospitalization, intensive care unit (ICU) admission], professional roles and wellbeing among ethnic minority and White HCWs. The cohort will study the impact on COVID-19 on physical and mental health of ethnic minority HCWs compared with White HCWs in the short and long term with consent for linkage with electronic health records for ≤25 years from the date of consent.

## Who is in the cohort?

Recruitment to the cohort began on 4 December 2020 and continued until 28 February 2021. In total, 17 891 HCWs from across the UK have been recruited into the study. Participants were considered eligible for the study if they were over the age of 16 years, lived and worked in the UK, and worked in health and social care or were a member of one of the UK healthcare regulators. This included ancillary workers such as cleaners and porters in healthcare settings. HCWs were invited to participate through two different channels. One was via an invitation from the various healthcare regulators and membership bodies within the UK, whereas the other was through a selection of National Health Service (NHS) trusts and health boards throughout the UK. A total of 12 280 participants were recruited through the first route, with 1018 participants recruited through the second route and the remaining 4593 recruited by visiting the study website directly or via social media.

A total of 1 052 875 e-mail invitations were sent by the healthcare regulators and membership bodies, summarized in Table 1. Healthcare regulators with large memberships such as the General Medical Council and the Nursing and Midwifery Council sent invitations to a representative sample of their cohorts, whereas smaller healthcare regulators sent invitations to their entire registers of healthcare workers. Twenty-eight NHS bodies consisting of NHS trusts in England, NHS regions in Scotland and NHS health boards in Wales (summarized in Table 2) engaged with their staff to increase recruitment, with invitations placed in trust-wide e-mails to all staff detailing recent events and news in each respective trust. Individual NHS trusts did not provide information on interactions with information related to the study in staff e-mails; however, on average, the response rate with the e-mails sent on behalf of the study by the healthcare regulators and membership bodies was 3.31%.

**Table 1:** Participating healthcare regulators and organizations in UK-REACH cohort

Partner abbreviation	Partner full name	Participants recruited (% of UK-REACH cohort)
GPhC	General Pharmaceutical Council	212 (1.2)
GMC	General Medical Council	3431 (19.2)
PSNI	Pharmaceutical Council of Northern Ireland	27 (0.2)
GOC	General Optical Council	344 (1.9)
NMC	Nursing and Midwifery Council	2391 (13.4)
HCPC	Health and Care Professionals Council	4963 (27.7)
GDC	General Dental Council	905 (5.1)
Serco	Serco	<10 (0.03)
Unknown	Not through any recruiting site	4593 (25.7)

Organizations who recruited <10 participants have had their numbers masked to reduce the risk of participant identification.

**Table 2:** Recruitment from National Health Service trusts and health boards into UK-REACH cohort

NHS trust abbreviation	Trust full name	Participants recruited (% of total cohort)
NH	Northumbria Healthcare NHS Foundation Trust	70 (0.4)
UHL	University Hospitals of Leicester	141 (0.8)
BH	Berkshire Healthcare NHS Foundation Trust	58 (0.3)
CRH	Chesterfield Royal Hospital NHS Foundation Trust	<10
SCAS	South Central Ambulance Service	18 (0.1)
SCNFT	Sussex Community NHS Foundation Trust	19 (0.1)
BCH	Bridgewater Community Health NHS Foundation Trust	<10
NHNFT	Nottinghamshire Healthcare NHS Foundation Trust	170 (1.0)
STNF	South Tees Hospitals NHS Foundation Trust	38 (0.2)
YDH	Yeovil District Hospital NHS Foundation NHS Trust	38 (0.2)
LAT	London Ambulance Service NHS Trust	11 (0.1)
DHNHFT	Derbyshire Healthcare NHS Foundation Trust	62 (0.3)
LG	Lewisham and Greenwich NHS Trust	25 (0.1)
UHSNFT	University Hospital Southampton NHS Foundation Trust	12 (0.1)
CLCH	Central London Community Healthcare NHS Trust	<10
RF	Royal Free London NHS Foundation Trust	<10
STGH	St George’s University Hospitals NHS Trust	26 (0.1)
LTHNFT	Lancashire Teaching Hospitals NHS Foundation Trust	135 (0.8)
STH	Sheffield Teaching Hospitals NHS Foundation Trust	30 (0.2)
BCHNFT	Birmingham Community Healthcare NHS Foundation Trust	<10
AC	Affinity Care	31 (0.2)
UHCW	University Hospitals Coventry and Warwickshire	18 (0.1)
BSMH	Birmingham and Solihull Mental Health NHS Foundation Trust	37 (0.2)
RBAH	Royal Brompton and Harefield NHS Foundation Trust	31 (0.2)
BLCHNFT	Black Country Healthcare NHS Foundation Trust	20 (0.1)
CDDFT	County Durham and Darlington NHS Foundation Trust	<10
NB	NHS Borders (Scotland)	<10
WHNT	Walsall Healthcare NHS Trust	<10

Trusts and health boards that recruited <10 participants have had their numbers masked to reduce the risk of participant identification.

Interested participants were directed to the cohort website (https://www.uk-reach.org) where they were able to provide contact details along with informed electronic consent, including permission to link to electronic healthcare records (EHRs) and to share pseudonymized research data with external researchers and to consent to participation in prize draws. The prize draw was offered to participants to incentivize completing individual questionnaires. Each prize draw consisted of 10 £250 Amazon gift vouchers, 10 £50 Amazon gift vouchers and 250 £5 Amazon gift vouchers, taken after each questionnaire period was closed. In order to be eligible for each prize draw, participants were required to complete the most recent questionnaire.

Table 3 shows the age, ethnicity and sex of those in the cohort compared with the age distribution of those in the NHS in England.^7^ The cohort shows a very similar age distribution to the NHS workforce, with an average age differential of 1 year.^8^ Whereas date of birth was captured during consent, ethnicity and sex were only captured by those who answered the baseline questionnaire, leading to variability in the amount of demographic information available from the cohort. The ethnicity information available, however, demonstrates that the UK-REACH cohort is more ethnically diverse than the NHS workforce with 26.7% of the UK-REACH cohort reporting a non-White ethnicity compared with 22.3% of the NHS workforce.^9^ Nevertheless, the study recruited fewer HCWs from White, Black and other ethnic groups than are present in the NHS workforce, whilst over-recruiting participants from Asian and mixed ethnic backgrounds. The UK-REACH cohort has a very similar sex balance as the NHS workforce, with 75.2% of the cohort female compared with 77% of the NHS.^7^

**Table 3:** Demographics breakdown for the UK-REACH cohort in comparison to the National Health Service workforce^1^^,^^8^

Variable	UK-REACH cohort (*n* = 17 891)	NHS workforce
**Age (years) (%)**		
<25	3	6
25–34	23	23
35–44	25	24
45–54	27	28
55–64	18	18
65+	3	2
**Sex (%)**	(*n* = 15 119)	
Male	24.6	23
Female	75.2	77
Prefer to use alternative term	0.1	N/A
Prefer not to answer	0.1	N/A
**Ethnicity (%)**	(*n* = 15 119)	
White	61.1	77.9
Black	3.9	6.5
Asian	17.2	11.3
Mixed	3.7	1.9
Other	1.9	2.6
Prefer not to answer/Not available	12.3	N/A

## How often have they been followed up?

Consented participants were asked to complete follow-up questionnaires 6 months (21 April–26 June 2021, *n* = 5632, response rate = 31.4% of consented participants) and 10 months (18 October–26 November 2021, *n* = 6535, response rate = 36.5% of consented participants) after the study opened for participants. These repeated topics from the baseline questionnaire, with minor adjustments to reflect the changes in the COVID-19 pandemic in the UK. Due to the unique pressures that the COVID-19 pandemic has placed upon healthcare workers, limiting the amount of time available for participants to complete questionnaires, each questionnaire was designed so that it could be either standalone or be used in a longitudinal arrangement.

During the follow-up questionnaires, ∼50% of participants who completed the baseline questionnaire had not completed a follow-up questionnaire. Participants who have not completed a follow-up questionnaire but have not withdrawn from the study are still considered not to be lost to follow-up, as they retain the ability to still be involved in the study. The ethnic diversity of the follow-up questionnaires varied slightly from the baseline questionnaire, with 26.7% of participants identifying as being from an ethnically diverse background at baseline compared with 28.7% (6-month follow-up questionnaire) and 27.5% (10-month follow-up questionnaire).

Between 18 October and 26 November 2021, participants were invited to provide consent to be sent a saliva sample kit to collect DNA data (*n* = 3976, response rate = 22.2% of consented participants). The samples were stored at the UK Biocentre (Milton Keynes, UK) after initial processing.

As UK-REACH is a UK-wide cohort study, no physical examinations of participants take place, with all interactions with participants conducted remotely via e-mail. Additional follow-up surveys have been planned for every 6 months until 2025, with questions based upon similar topics to those already used and additional questions to provide insight into novel research questions.

## What has been measured?

After consenting to join the cohort, participants were invited to complete the baseline questionnaire, which addresses a range of topics related to COVID-19, leading to a wide range of qualitative and quantitative data being collected.

In addition, participants gave permission to use data from their EHRs for a period of 25 years from the date of consent, allowing longitudinal tracking of the effect of the pandemic on participants’ health. Not all participants who consented to have their EHRs linked completed the baseline questionnaire due to the consent process and the questionnaires being discrete options for participants.

Table 4 provides an overall summary of the data available from the cohort, whereas additional information on the questions asked, response options and question sources can be found in the UK-REACH data dictionary (https://www.uk-reach.org/data-dictionary). In brief, questions included information about physical and mental wellbeing based upon the General Practice physical activity questionnaire (GPPAQ), EQ-5D and the post-traumatic stress disorder checklist—civilian version (PCL-C). Harassment and discrimination was addressed via questions from the Everyday Discrimination Scale, the NHS Staff survey and the Understanding Society cohort questionnaires. Trait and psychological measures were measured using questions from the Understanding Society cohort questionnaires, a brief version of Levenson’s Locus of Control Scale and fatalism questions from Shen *et al.*^10^ Additional pertinent questions were designed by the UK-REACH study team.

**Table 4:** Summary of data collected at each phase^1^

Phase (dates) (*n*)	Topics
Baseline questionnaire (December 2020–February 2021) (*n* = 15 471)	Ethnicity
	Nationality, religion and languages
	Other demographics and education
	Work
	Home and social life
	Harassment and discrimination
	Physical and mental health, wellbeing
	COVID-19 experiences and beliefs
	Trait and state psychological measures
	Open-ended questions
	Questionnaire evaluation questions
First follow-up questionnaire (April 2021–June 2021) (*n* = 5632)	Ethnicity
	Nationality, religion and languages
	Other demographics and education
	Work
	Home and social life
	Harassment and discrimination
	Physical and mental health, wellbeing
	COVID-19 experiences and beliefs
	Long COVID
	Vaccine symptoms
	Trait and state psychological measures
	Open-ended questions
	Questionnaire evaluation questions
Second follow-up questionnaire (October 2021– November 2021) (*n* = 6535)	Ethnicity
	Nationality, religion and languages
	Other demographics and education
	Work
	Home and social life
	Harassment and discrimination
	Physical and mental health, wellbeing
	COVID-19 experiences and beliefs
	Long COVID
	Vaccine symptoms
	Trait and state psychological measures
	Open-ended questions
	Questionnaire valuation questions
Saliva testing (*n* = 3976)	DNA data
Linkage to electronic healthcare records (ongoing)	COVID-19 clinical outcomes (acute infection, antibody status)
	Co-morbidities
	Patterns of healthcare usage

## What has it found?

Data sets collected from the cohort have contributed to multiple outputs, providing insight into HCWs during the COVID-19 pandemic, and a full list of these can be found at https://www.uk-reach.org/publications.

### Vaccine hesitancy in HCWs

An analysis of the drivers of vaccine hesitancy was the first major finding from the UK-REACH cohort.^11^ This analysis of interim data collected from 4 December 2020 to 19 February 2021 included 11 584 HCWs, of whom 23% reported vaccine hesitancy. HCWs from Black Caribbean (54.2% reported hesitancy), Mixed White and Black Caribbean (38.1%), Black African (34.4%), Chinese (33.1%), Pakistani (30.4%) and White Other (28.7%) ethnic groups were significantly more likely to be hesitant when compared with White British HCWs (21.3% hesitant). The following factors were also significant in predicting hesitancy towards the COVID-19 vaccine: younger age, female sex, higher score on a COVID-19 conspiracy beliefs scale, lower trust in employer, lack of influenza vaccine uptake in the previous season, previous COVID-19 and pregnancy. Qualitative analysis of a smaller selection of HCWs (*n* = 99) from a separate work package of the UK-REACH project, who participated in face-to-face interviews and focus groups revealed a range of reasons that HCWs were hesitant about the COVID-19 vaccines. Reasons provided as contributors to vaccine hesitancy included: lack of trust in government and employers, safety concerns due to the speed of vaccine development, lack of ethnic diversity in vaccine studies, and confusing and conflicting information. Qualitative analysis also provided some strategies for addressing vaccine hesitancy in ethnic minority HCWs, such as inclusive communication, involving HCWs in the vaccine rollout and promoting vaccination through trusted networks.

### Infection risk in HCWs

HCWs, particularly those from ethnic minorities, have been shown to be at higher risk of infection with SARS-CoV-2 than the general population, although evidence is conflicted about the predictors and mediating factors of infection in HCWs.^12^ Analysis of 10 772 HCWs who reported working during the first UK national lockdown in March 2020 revealed that 2496 (23.2%) had some evidence of previous SARS-CoV-2 infection (via polymerase chain reaction tests, serology testing or self-reported COVID-19 diagnosis). Statistical analyses of the baseline UK-REACH survey revealed that demographic factors such as younger age and high religiosity were associated with an increased infection risk. A range of occupational factors were also associated with increased infection risk: attending to a higher number of SARS-CoV-2-positive patients, working in a nursing role (compared with a medical role), lack of access to personal protective equipment (PPE) and working in an ambulance setting. HCWs working in an ICU and those who worked in the south-east of England or Scotland were at lower risk of infection (when compared with the West Midlands of England as a reference group). Black ethnic groups were initially identified as being at higher risk but adjusted statistical models revealed factors that mediated the elevated infection risk.^13^

### PPE access for HCWs

Access to PPE may prevent transmission of SARS-CoV-2 and anecdotal reports exist of a lack of access to PPE by HCWs.^14^^,^^15^ Two analyses were undertaken to examine the factors relating to PPE access for HCWs in the UK. The primary analysis included participants who answered baseline questions about access to PPE during the first UK national lockdown (23 March 2020) (*n* = 10 508), whereas the secondary analysis included those who answered baseline questions about PPE access during the baseline questionnaire period (4 December 2020–28 February 2021) (*n* = 12 252). The primary analysis found that only 35.2% of HCWs reported being able to access appropriate PPE all of the time during the first UK national lockdown, whereas the secondary analysis found that 83.9% of HCWs had access to PPE all of the time during the baseline questionnaire period.^16^ Several factors predicted access to PPE in both analyses, such as age (being older predicted greater access to PPE), being Asian (vs White) and role (allied health professionals, dentists and those who saw the most COVID-19 patients were all predictors of reduced access to PPE all the time). Both analyses also showed that access to PPE was not uniform across the UK, as those in south-west and north-west England were able to access PPE more frequently than those in London. In summary, access to PPE for HCWs was particularly limited during the first lockdown and access varied based on socio-demographic, occupational and geographic factors.^16^

## What are the main strengths and weaknesses?

The UK-REACH study is a UK-wide study, capturing information from the wide range of roles that form healthcare services in the UK, including ancillary workers who are often not included in such studies. The involvement of the healthcare regulators, NHS trusts and health boards, and various membership bodies has enabled the study to recruit from a large pool of HCWs, providing a diverse and representative sample of the wide range of clinical-based job roles within the UK healthcare sector. However, some staff such as porters, cleaners and kitchen staff are under-represented in the cohort, despite a targeted approach to recruit from these groups in collaboration with Serco, a UK public services provider, who are routinely contracted to provide ancillary staff in healthcare sites across the UK. The lack of representation in the cohort from groups with lower socio-economic status may cause findings to under-report the effects of outcomes on those groups.

A significant strength of the UK-REACH study is the ethnic diversity of the cohort, with 26.7% identifying with an ethnic minority background, particularly as ethnic minorities are often under-represented in studies.^17^ Nevertheless, Black ethnic groups remain under-represented in the UK-REACH study, which should be a key target for future studies of both COVID-19 and HCW occupational health with learnings from the UK-REACH study made available to facilitate this. In future, the high percentage of ethnic minority HCWs present in the cohort will allow wider research questions to be asked outside the current COVID-19 focus.

It is likely that the effects of the pandemic have placed additional strains on HCWs of all ethnicities for an extended period, which may have limited study participation, possibly because participants do not have time or do not wish to answer large numbers of questions about how the pandemic has affected them.

The online-only nature of the UK-REACH study enabled recruitment of participants across the UK, giving a national picture of the impact of the COVID-19 pandemic on HCWs of varying ethnicities. However, the exclusive use of digital communication methods (e.g. e-mail and social media) to advertise the study and digital data collection may have limited participation in the study, particularly amongst certain staff groups such as those without access to a computer routinely throughout. As result, the study is likely to contain biases due to participant self-selection. Initial recruitment was maximized via repeated communications from healthcare regulators and NHS trusts, with many participants receiving invitations to participate from both their regulator and their employer at different times. Reminder e-mails were also sent by the study team to participants who had registered their interest with the study by creating an account on the study website but had not completed the consent process, and to participants who had consented to the study but had not completed the baseline questionnaire. For the follow-up questionnaires, consented participants were contacted to invite them to fill in the questionnaires, with reminders to participants to encourage completion.

## Can I get hold of the data? Where can I find out more?

The cohort website (https://www.uk-reach.org) contains an up-to-date record of all research activities, including publications in peer-reviewed journals, pre-print articles and other related study news.

Participants have consented to their pseudonymized data being made available to other approved researchers and we welcome requests for collaboration and data access. Access to the resource requires completion of a proposal form, including a lay summary of the proposed research. Applications to access the resource will be assessed for consistency with the data access policy by the Scientific Committee, which has participant representation. Interested researchers are encouraged to contact the study management team and principal investigator Professor Manish Pareek via uk-reach@leicester.ac.uk. Access to forms and more detail on the process can be found at https://www.uk-reach.org/data_sharing.

## Notes

UK-REACH Collaborative group: ^*^Manish Pareek (Chief investigator), Laura Gray (University of Leicester), Laura Nellums (University of Nottingham), Anna L Guyatt (University of Leicester), Catherine John (University of Leicester), I Chris McManus (University College London), Katherine Woolf (University College London), Ibrahim Akubakar (University College London), Amit Gupta (Oxford University Hospitals), Keith R Abrams (University of York), Martin D Tobin (University of Leicester), Louise Wain (University of Leicester), Sue Carr (University Hospital Leicester), Edward Dove (University of Edinburgh), Kamlesh Khunti (University of Leicester), David Ford (University of Swansea), Robert Free (University of Leicester).

## Ethics approval

The study was approved by the Health Research Authority (Brighton and Sussex Research Ethics Committee; ethics reference: 20/HRA/4718). All participants gave electronic informed consent. Trial ID: ISRCTN11811602.

## Data Availability

See ‘Can I get hold of the data?’ above.
